# CD8^+^ T-cell cross-competition is governed by peptide–MHC class I stability

**DOI:** 10.1002/eji.201142010

**Published:** 2011-11-28

**Authors:** Ian Galea, Jana Stasakova, Melanie S Dunscombe, Christian H Ottensmeier, Tim Elliott, Stephen M Thirdborough

**Affiliations:** Cancer Sciences Unit, University of Southampton, Faculty of Medicine, Southampton General HospitalSouthampton, UK

**Keywords:** CD8^+^ T cells, Competition, Immunodominance, MHC, Peptide

## Abstract

A major contributing factor to the final magnitude and breadth of CD8^+^ T-cell responses to complex antigens is immunodomination, where CD8^+^ T cells recognizing their cognate ligand inhibit the proliferation of other CD8^+^ T cells engaged with the same APC. In this study, we examined how the half-life of cell surface peptide–MHC class I complexes influences this phenomenon. We found that primary CD8^+^ T-cell responses to DNA vaccines in mice are shaped by competition among responding CD8^+^ T cells for nonspecific stimuli early after activation and prior to cell division. The susceptibility of CD8^+^ T cells to ‘domination’ was a direct correlate of higher kinetic stability of the competing CD8^+^ T-cell cognate ligand. When high affinity competitive CD8^+^ T cells were deleted by self-antigen expression, competition was abrogated. These findings show, for the first time to our knowledge, the existence of regulatory mechanisms that direct the responding CD8^+^ T-cell repertoire toward epitopes with high-stability interactions with MHC class I molecules. They also provide an insight into factors that facilitate CD8^+^ T-cell coexistence, with important implications for vaccine design and delivery.

## Introduction

Despite the presence of high numbers of potential epitopes within a complex immunogen, the elicited CD8^+^ T-cell response rapidly becomes skewed toward a few epitopes 1. These CD8^+^ T-cell determinants can be broadly divided into two categories, immunodominant epitopes (IDEs) and subdominant epitopes (SDEs), based upon the relative magnitude of the CD8^+^ T-cell populations induced. Such a restricted pattern of T-cell responses in terms of target specificity favors the emergence of viral escape mutants and limits the effectiveness of CD8^+^ T-cell-based genetic vaccines 2–5. The mechanisms behind immunodominance are multifold and include differences in CD8^+^ T-cell precursor frequency, epitope abundance on the priming APCs, and competition between CD8^+^ T cells for activation 1, 6. This study focuses on the last factor, defined as immunodomination, and asks how the kinetic stability of peptide–MHC (pMHC) class I complexes influences simultaneous, competing CD8^+^ T-cell responses to unrelated epitopes.

While it is agreed that CD8^+^ T cells of the same epitope specificity can compete for their cognate ligand when expressed by a shared APC, it remains controversial whether competition between CD8^+^ T cells of different epitope specificity affects priming and immunodominance 7. In experiments involving the adoptive transfer of TCR-transgenic CD8^+^ T cells, several studies have shown that host CD8^+^ T cells of different specificity compete for APCs upon antigen challenge 8, 9, whereas others have not observed this ‘cross-competition’ phenomenon 10, 11. In silico modeling has suggested that differences in epitope abundance and differing TCR avidities could account for some of the controversy. Scherer and colleagues 12 found that at low epitope expression levels per APC, CD8^+^ T cells compete for access to their cognate ligand, so there is competition between CD8^+^ T cells of the same but not different epitope specificity. At high epitope expression levels, however, CD8^+^ T cells compete for a nonspecific stimulus, namely, access to the APC surface, which is a shared resource.

The half-life of pMHC class I complexes is a key parameter that affects epitope density on the priming APC surface and subsequent CD8^+^ T-cell responses 13, 14. Peptides with slow dissociation rates show increased resistance to tapasin editing during ER loading onto MHC class I molecules, leading to higher initial epitope densities on the APC 15, 16. Furthermore, because of their inherent high stability, these pMHC complexes may persist at the cell surface during APC migration to draining lymph nodes and also promote prolonged CD8^+^ T cell–APC encounters 17, 18, perhaps resulting in preferential CD8^+^ T-cell responses 18. There is evidence from studies investigating the mechanisms of immunodominance that during competitive CD4^+^ T-cell responses, peptides with low-stability interactions with MHC class II molecules lose the ability to sustain T-cell expansion 19. However, it is not known whether pMHC stability contributes to CD8^+^ T-cell competition, and if this property can be exploited to broaden responses to polyepitope vaccines. To this end we employed a model system based in part on analyses by Mylin et al. 20, who characterized the B6 (H-2^b^) response hierarchy to SV40 large T antigen (Tag). They identified three IDEs (designated T1, T2/3, and T4), and one SDE (designated T5) that elicits a CD8^+^ T-cell response in the absence of the IDEs. Here we have measured endogenous CD8^+^ T-cell responses to the subdominant T5 (D^b^-restricted) epitope when coexpressed with kinetic-stability variants of the T4 (K^b^-restricted) epitope following immunization with plasmid DNA vectors in normal or Tag-tolerant mice. Our results demonstrate that the ability of competitive CD8^+^ T cells to exert immunodomination directly correlates with pMHC stability.

## Results

### SV40 Tag immunodominance hierarchy

In the C57BL/6 mouse, immunization with SV40 Tag-transformed cells or a recombinant vaccinia virus encoding full-length Tag induces a CD8^+^ T-cell response directed against three H-2D^b^-restricted peptides, designated epitopes T1, T2/3, and T5, and one H-2K^b^ epitope T4 in the hierarchical order T4>T1>T2/3>>T5 20. Competitive CD8^+^ T-cell responses have typically been observed in secondary responses, when the ratio of T cells to antigen is high 21. To determine whether CD8^+^ T-cell responses induced by DNA vaccination showed similar patterns of immunodominance, C57BL/6J mice were immunized by intramuscular injection with plasmid DNA encoding amino acids 205–566 of Tag (pΔTag). This truncated Tag sequence encodes the four defined CD8^+^ T-cell epitopes but for safety excludes p53 and Rb protein-binding sites 22. Groups of mice were also immunized with plasmid DNA encoding the nominal T1, T4, or T5 epitopes fused immediately 3′ to the N-terminal domain of Fragment C (DOM) from tetanus toxin. This vaccine design, referred to as pDOM-epitope, allowed comparison of response magnitudes to the individual CD8^+^ T-cell determinants in the absence of known competitive H-2^b^ epitopes 23. After 12 days, the number of peptide-specific CD8^+^ T cells induced was measured directly ex vivo by IFN-γ ELISPOT.

**Figure 1:**
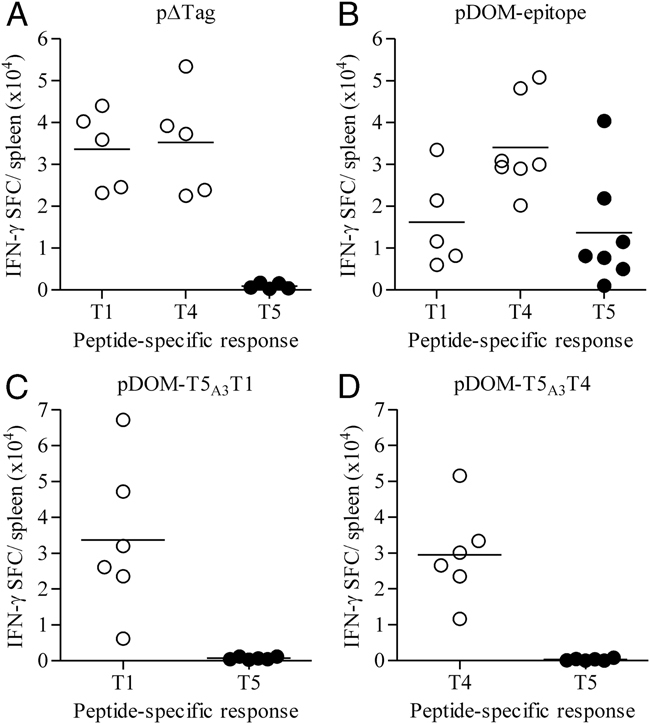
SV40 Tag immunodominance hierarchy in WT mice primed by DNA vaccination. C57BL/6J mice were immunized with (A) pΔTag, (B) pDOM-epitope encoding the individual Tag-derived determinants as indicated, (C) pDOM-T5_A3_T1 or (D) pDOM-T5_A3_T4. After 12 days, the number of epitope-specific T cells induced was assayed by IFN-γ ELISPOT. The results shown are combined from two separate experiments, with each data point representing an individual mouse. Horizontal bars depict group means.

Immunization with pΔTag elicited an effector T-cell repertoire largely focused on epitopes T1 and T4 (Fig. 1A). Epitope T5-specific responses were barely detectable above background (mean 43 spot forming cells (SFCs)/10^6^ splenocytes compared to 15 with an irrelevant peptide). When the determinants were delivered individually via pDOM-epitope, intermediate responses were obtained to epitope T5 (mean 915 SFCs/10^6^ splenocytes; Fig. 1B). In addition, the T1-specific response was smaller in magnitude than that elicited by pDOM-T4 immunization (Fig. 1B). Hence, the subdominant status of T5 was lost when the epitope was expressed alone, in the context of DOM as opposed to ΔTag.

### Inter- and intra-allelic competition shapes primary CD8^+^ T-cell responses to DNA vaccines

To examine whether co-expression of an IDE with T5 would re-establish the response hierarchy, we generated a plasmid DNA construct encoding the H-2D^b^-restricted epitopes T1 and T5 separated by a 3-alanine spacer, appended in tandem to the C-terminus of DOM. Immunization with this vaccine elicited a primary CD8^+^ T-cell response that focused only on epitope T1 (Fig. 1C); moreover, when T5 was co-expressed with the H-2K^b^-restricted epitope T4, a similar phenomenon was observed (Fig. 1D). Since the latter epitope is presented by a different class I allomorph, competition for binding to the MHC could be excluded as a possible cause of the observed immunodominance. The apparent ability of IDE-reactive T cells to impede the simultaneous expansion of T5-specific T cells suggested that primary response patterns to DNA vaccines are indeed shaped by immunodomination.

### Selective thymic tolerance of IDE-reactive CD8^+^ T cells ablates competition

**Figure 2:**
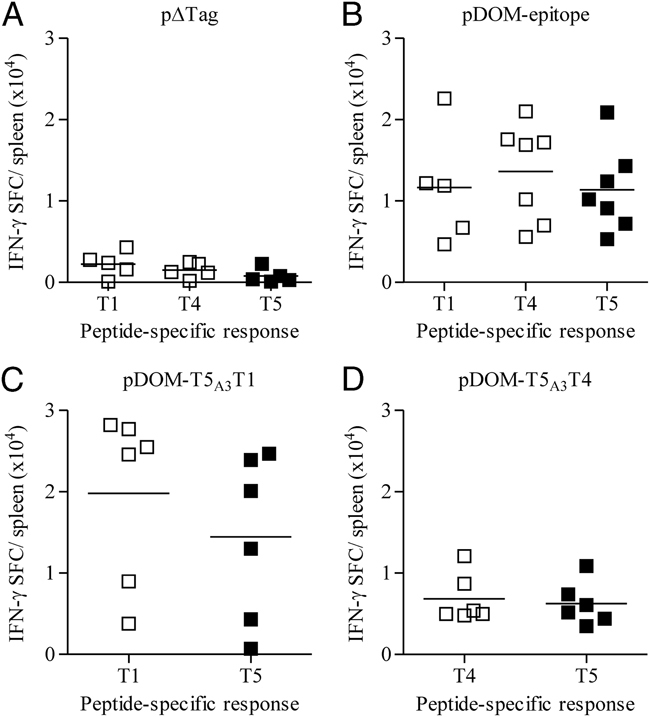
SV40 Tag immunodominance hierarchy in TRAMP mice primed by DNA vaccination. TRAMP mice were immunized with (A) pΔTag, (B) pDOM-epitope encoding the individual Tag-derived determinants as indicated, (C) pDOM-T5_A3_T1 or (D) pDOM-T5_A3_T4. After 12 days, the number of epitope-specific T cells induced was assayed by IFN-γ ELISPOT. The results shown are combined from two separate experiments, with each data point representing an individual mouse. Horizontal bars depict group means.

To test the possibility that the observed subdominance of epitope T5 was due to T-cell competition, we asked whether reducing the precursor frequency of high-affinity IDE-reactive T cells would liberate T5-specific responses from domination. For these experiments, we utilized female TRAMP (transgenic adenocarcinoma of the mouse prostate) mice transgenic for SV40 Tag 24. In this model, Celis and colleagues have demonstrated that high-affinity T cells specific for T4 are tolerized by thymic deletion, whereas CD8^+^ T cells with high affinity for T5 survive negative selection 25. In these mice, and consistent with the findings of Grossmann et al. 25, self-tolerance was found to limit the responsiveness of CD8^+^ T cells specific for each of the IDE when immunized with pΔTag (Fig. 2A cf. 1A). However, vaccination with pDOM-epitope expanded T1, T4, or T5 specific cells to levels comparable to those seen in WT mice (Fig. 2B cf. 1B). The functional avidity of these effector populations was assessed by using serial peptide concentrations and defining the SD_50_ as the peptide concentration yielding half-maximal counts in the IFN-γ ELISPOT assay. In TRAMP versus WT mice (Supporting Information Fig. 1), the SD_50_ of T1 or T4 reactive T cells differed by 38- and six-fold, respectively, whereas peptide concentrations required to induce the half-maximal number of IFN-γ-producing T5-specific cells differed only three-fold. These data show that CD8^+^ T cells specific for T5 are less susceptible to tolerogenic pressure 25–28 and confirm earlier observations that low avidity IDE-specific CD8^+^ T cells can be expanded from a tolerized repertoire when foreign cognate CD4^+^ T-cell help is provided 29.

Using the TRAMP model, we next asked whether the residual repertoire of lower-avidity T1 or T4 specific CD8^+^ T cells would still dominate responses when their cognate epitope ligand was delivered in tandem with epitope T5 (via pDOM-T5_A3_T1 or pDOM-T5_A3_T4). In these Tag-tolerant mice, dominance was abrogated, with both vaccines eliciting coexistent effector populations (Fig. 2C and D). Similar patterns of responses to pDOM-T5_A3_T4 immunization were observed when T5/D^b^- and T4/K^b^-tetramer binding, rather than IFN-γ ELISPOT analysis was used to quantitate T-cell priming (data not shown). These findings suggest that responses to the T5 epitope are inhibited by the immunodominant T4 response in non-transgenic mice. However, when the IDE-specific repertoire is subject to tolerance, hierarchical control by the T1 or T4 epitope is broken, and T5-specific responses are elicited. The reduced susceptibility of the SDE T5 to tolerance induction likely reflects the relatively short half-life of T5/D^b^ complexes 20, 30, resulting in diminished steady-state epitope presentation levels and a failure to induce negative selection 27. Moreover, when activated in the periphery, low epitope density may render the T5-specific preimmune repertoire vulnerable to domination.

### Domination by IDE-reactive CD8^+^ T cells correlates with pMHC stability

**Figure 3:**
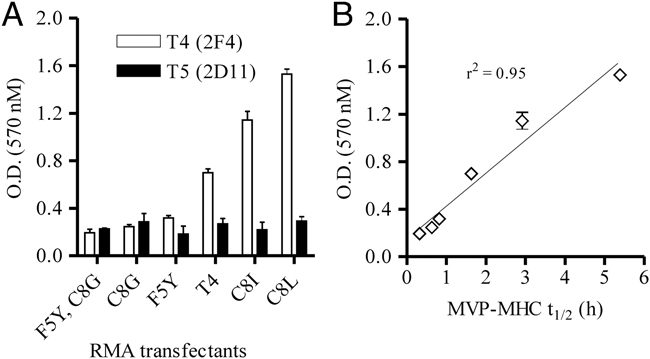
In vitro recognition of epitope T4 kinetic-stability variants and correlation with pMHC stability. RMA cells were electroporated with empty vector or pDOM-T5_A3_T4 constructs, encoding each of the T4 MVPs, and used to stimulate T-cell hybridomas raised against native T4/K^b^ (2F4) or T5/D^b^ (2D11) complexes. (A) Induction of β-gal activity was measured after 18 h using the substrate chlorophenol red-b-D-galactopyranoside. Each data point represents the mean for three wells (minus mean background obtained with empty vector transfectants), and error bars represent the SD. The results shown are representative of three separate experiments. (B) Recognition of epitope T4 kinetic-stability variants correlated with pMHC stability.

To establish the relationship between T-cell competition and the kinetic stability of pMHC-complexes, we generated a series of pDOM-T5_A3_T4 constructs encoding amino acid substitutions in the anchor residues of the T4 octamer. Initially, the half-life for dissociation of these MHC variant peptides (MVPs) from H-2K^b^ at the cell surface was determined as a relative measure of the stability of the pMHC complex on the priming APC 15. The half-life for dissociation of these T4 MVPs and epitope T5 are listed in Table 1, together with their capacity to stimulate CD8^+^ T cells elicited by pDOM-T4 vaccination. Responses were measured to the native T4 epitope and to each of the peptide variants, and we observed no difference in the relative number of IFN-γ-producing cells when the MVPs were present at saturation (Table 1). Relative levels of presentation of the T4 kinetic stability variants and T5 were assessed in vitro by electroporating Rauscher virus-induced H-2^b^ lymphoma (RMA) cells with each of the pDOM-T5_A3_T4 MVP constructs. Induction of β-galactosidase activity by T-cell hybridomas 2F4 and 2D11, raised against the native K^b^/VVYDFLKC and D^b^/QGINNLDNL complexes, respectively, was used as an indicator of the ability of the RMA cells to process and present the class I determinants from DOM. Each of the kinetic stability variants of epitope T4 was recognized by the T-cell hybridoma 2F4 (Fig. 3A), with overall activation correlating with the half-life for dissociation (Spearman: *r*=1.00 and *p*=0.023; Fig. 3B). Similarly, the T5-specific T-cell hybridoma was stimulated at a low, but detectable (∼eight-fold higher than background), level by each of the pDOM-T5_A3_T4 MVP transfectants (Fig. 3A), and compatible with the short half-life of T5/D^b^ complexes.

**Table 1:** Half-life of dissociation of epitope T4 and its variants, together with their capacity to stimulate epitope T4-specific T_CD8_

Peptide	Sequence	*t*_1/2_ (h)	Percentage of T4-specific response (1 μM)
T4	VVYDFLKC	1.6	100
C8L	VVYDFLKL	5.4	91
C8I	VVYDFLKI	2.9	91
F5Y	VVYDYLKC	0.8	100
C8G	VVYDFLKG	0.6	100
F5Y, C8G	VVYDYLKG	0.3	85
T5	QGINNLDNL	0.9	

**Figure 4:**
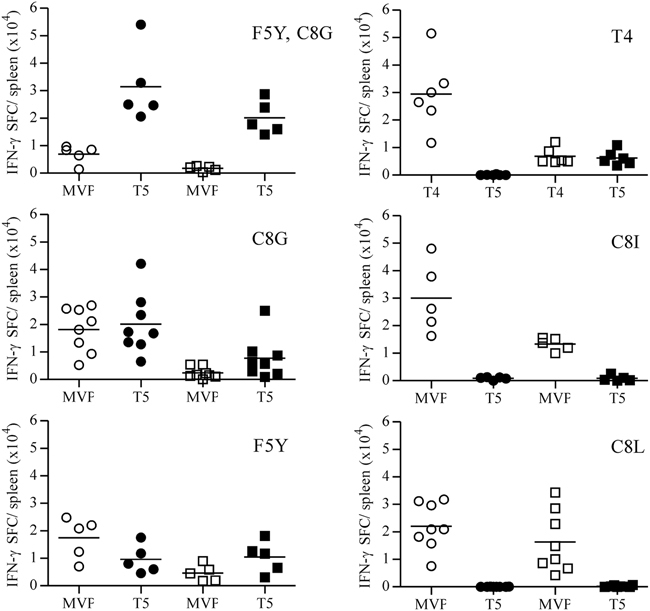
Immunogenicity of epitope T4 kinetic-stability variants and dominance over T5-specific CD8^+^ T-cell responses. Non-transgenic (circles) and TRAMP (squares) mice were immunized with pDOM-T5_A3_T4, encoding the T4 MVPs indicated on the top right of each panel. After 12 days, the number of T4 (open) and T5 (closed) epitope-specific CD8^+^ T cells induced were assayed by IFN-γ ELISPOT, using native peptides for the restimulation. The results shown are combined from two separate experiments, with each data point representing an individual mouse.

**Figure 5:**
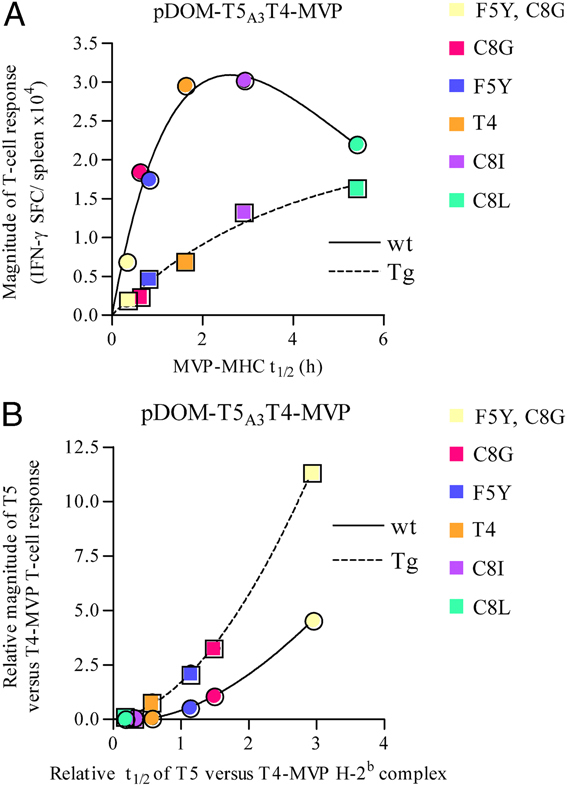
Correlations of pMHC stability with immunogenicity and CD8^+^ T-cell co-existence. (A) The magnitude of the T4 MVP-specific response versus the kinetic stability of the variant peptides. (B) The ratio of the T5 to T4 MVP population size versus the ratio of T5 to T4 MVP-kinetic stability. All the data points are shown in Table 1 and Fig. 4. Curves were fitted by GraphPad Prism 4 software using non-linear regression and one phase exponential association.

To evaluate the immunogenicity of the kinetic-stability variants relative to the native T4 epitope, and the capacity of subsequent responses to dominate T5, we immunized WT or TRAMP mice with the pDOM-T5_A3_T4 vaccines encoding the T4 anchor residue substitutions. Each peptide variant expanded T4-specific T cells in WT and TRAMP mice (Fig. 4). With the exception of WT responses to the slow off-rate variant C8L, the size of the effector populations correlates with pMHC stability (Fig. 5A). Similarly, there is a consistent relationship between the ability of MVP responses to dominate T5 and relative pMHC half-lives of epitope T5 versus T4-MVP (Spearman: *r*=0.96 and *p*=0.023 in WT mice, and *r*=0.98 and *p*=0.001 in TRAMP mice; Fig. 5B). Thus, highly stable variants elicited similar or enhanced competitive T4 responses, whereas variant epitopes with rapid off-rates from H-2K^b^ showed diminishing competitive responses, culminating in a reversal of the immunodominance hierarchy with epitope F5Y, C8G (Fig. 4).

We have reported previously that prolonged presentation of high stability pMHC complexes via pDOM-epitope delivery limits the magnitude of primary CD8^+^ T-cell responses, possibly due to supra-optimal stimulation leading to clonal deletion 16. This previous finding is not inconsistent with the poor correlation between the slow off-rate of epitope C8L and the magnitude of the CD8^+^ T-cell response in WT mice (Fig. 5A).

### Competition occurs early after CD8^+^ T-cell activation

**Figure 6:**
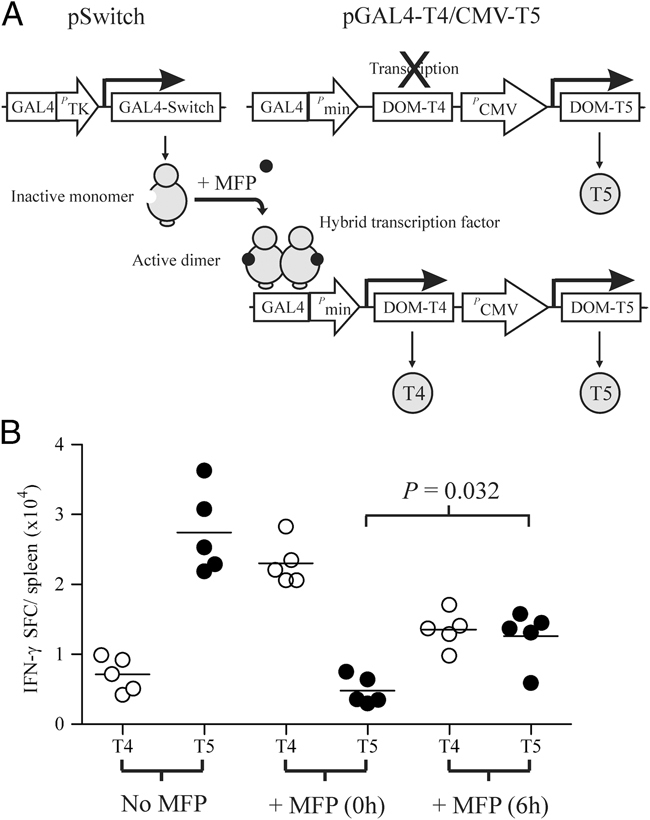
Domination of epitope T5-specific CD8^+^ T cells occurs early after activation. (A) Representative structure of pGAL4-T4/CMV-T5 and schema of the modified GeneSwitch system used to temporally regulate T4 expression relative to uncontrolled T5 expression. (B) C57BL/6J mice were immunized with pGAL4-T4/CMV-T5 along with pSwitch, and left untreated or administered with 5 mg/kg MFP at the time points indicated. The number of peptide-specific T cells elicited was determined 12 days later by IFN-γ ELISPOT. The bars indicate group medians and results are representative of two separate experiments. *p*-Values were determined by the non-parametric Mann–Whitney test (two-tailed).

Finally, we investigated the time frame of CD8^+^ T-cell competition using a mifepristone (MFP)-responsive gene regulation system (GeneSwitch™ 31) to temporally regulate T4 epitope expression relative to constitutive T5 epitope expression (shown schematically in Fig. 6A). C57BL/6J mice were co-injected with pGAL4-T4/CMV-T5 and pSwitch, and left untreated or administered a 5 mg/kg dose of MFP at 0 or 6 h post-immunization. Importantly, MFP at this dose does not affect the magnitude of an endogenous response to pDOM-epitope vaccination 32. In the absence of MFP, basal leakiness of T4 transgene expression primed a T4-specific response that was lower in magnitude than that induced by uncontrolled, CMV promoter-driven T5 epitope expression (Fig. 6B cf. 1B). Conversely, when MFP was given immediately after DNA immunization, the elicited CD8^+^ T-cell response focused on epitope T4, with diminished reactivity to epitope T5. However, when the inducer was given 6 h after immunization, the magnitude of T5-specific responses approximated those to T4 (Fig. 6B). These data suggest that cross-competition occurs early after initial CD8^+^ T-cell activation and prior to cell division.

## Discussion

This report demonstrates that primary CD8^+^ T-cell immunodominance hierarchies to DNA vaccines are shaped by competition among CD8^+^ T cells of different pMHC specificities, for a common stimulus, early after activation and prior to cell division. We show for the first time that the susceptibility of CD8^+^ T cells to domination is a direct correlate of higher kinetic stability of the competing CD8^+^ T-cell cognate ligand. In tolerant mice, an affinity advantage for the SDE-specific CD8^+^ T-cell population compensated for lower epitope density and facilitated coexistence. These findings are compatible with immunodominant CD8^+^ T cells in WT mice having a stimulation threshold advantage at priming, with pMHC class I stability regulating the magnitude of TCR engagement 17.

Previous studies on T-cell competition have shown that CD8^+^ T cells of different epitope specificity can compete with each other when epitopes are presented on shared APCs 33–36, and when the ratio of CD8^+^ T cells to APCs is high 8, 36–38. It has been suggested that the resource that CD8^+^ T cells compete for is access to the APC itself or APC-derived factors, such as costimulatory molecules or cytokines 8. At this time we cannot distinguish between these possibilities. However, our data together with a recent report investigating CD4^+^ T-cell immunodominance hierarchies 19 underline the importance of the kinetic stability of pMHC complexes in determining the final specificity of T-cell responses. Sant and colleagues found that peptides with fast dissociation rates from I-A^d^ initially expand CD4^+^ T cells, but fail to sustain their activation throughout the course of the response 19. Importantly, this failure only manifested when the short half-life epitope was co-administered with an IDE. Thus, for both CD8^+^ and CD4^+^ T-cell responses there exist regulatory mechanisms independent of antigen processing and editing effects, which direct the responding T-cell repertoire toward epitopes with high-stability interactions with MHC molecules.

In contrast to the findings of Sant 19, our model requires the IDE to be expressed in the first 6 h following DNA delivery in order for competition to occur. This time scale is similar to that previously reported after adoptive transfer experiments, in which competing TCR transgenic CD8^+^ T cells needed to be present in the first few hours to interfere with endogenous CD8^+^ T-cell responses 38. These findings suggest that competition occurs at the stage of CD8^+^ T-cell recruitment into the response. Experiments using multiphoton intravital microscopy to describe initial, synchronized CD8^+^ T-cell-DC encounters in lymph nodes have shown that CD8^+^ T-cell activation occurs in three successive phases 39. During phase one, CD8^+^ T cells undergo multiple transient encounters with DCs over several hours. This is followed by a second period lasting 8–20 h, during which CD8^+^ T cells form stable interactions with DCs and begin to secrete cytokines. The third phase coincides with the onset of proliferation and is characterized by the return of high CD8^+^ T-cell mobility and brief contacts with DCs. A recent report has shown that the duration of phase one correlates inversely with the number of complexes of cognate pMHC per DC 17. It is therefore possible that CD8^+^ T cells responding to high or low stability pMHC complexes differ with respect to the time required for the transition from phase one to phase two 18. If the number of sites on an APC that can form stable interactions with CD8^+^ T cells are limiting, perhaps CD8^+^ T cells compete for progression to phase two. CD8^+^ T cells interact with DCs by way of bundles of microvilli on the DC surface 40. The concentration of pertinent pMHC complexes and costimulatory molecules in these distinct membrane structures culminates in multifocal synapse assembly and stable DC–CD8^+^ T-cell interactions. Of relevance to immunodomination, the surface area of the DC committed to microvilli formation is limited and is likely related to DC cytoskeletal polarization during CD8^+^ T-cell activation 40. Thus, it is possible that by monopolizing DC polarization, dominant CD8^+^ T cells prevent the coordinated delivery of signals from antigen, adhesion/costimulatory molecules, and soluble mediators to SDE-specific CD8^+^ T cells.

In terms of vaccine development, targeting epitopes that form low stability complexes with MHC molecules may provide a strategy to bypass tolerance to tumor self-antigens 25, 28. However, the sensitivity of these low stability determinants to domination makes the development of improved vectors that incorporate minimal antigenic baggage, and that do not entail complex administration schedules 41, critical.

## Materials and methods

### Plasmids

To construct the pΔTag vector, cDNA encoding amino acids 205–566 of Tag flanked by *Not I* and *Xma I* restriction sites was amplified by RT-PCR from 293-T cells and cloned into pCI-E3/19K 42. The pDOM-epitope vaccines encoding the nominal Tag-derived CD8^+^ T-cell determinants were constructed as previously described 42. To construct the MFP-responsive DOM-T4 expression vector, an ORF flanked by *Hind III* and *Pme I* restriction sites was amplified by PCR using pDOM-T4 as template and cloned into *pGene/V5* (Invitrogen, Carlsbad, CA). The GAL4/DOM-T4/BGHpA expression cassette was then PCR amplified and inserted into pDOM-T5 as a *Bgl II* fragment to generate the construct pGAL4-T4/CMV-T5. Plasmid DNA was purified for immunization using a QIAfilter Mega kit (Qiagen, Hilden, Germany). All cDNAs were verified by sequencing.

### Brefeldin A decay assay

RMA-S cells were cultured overnight at 26°C in serum-free media (X-VIVO 15, Lonza, MD) and then for 60 min in the presence of BreFeldinA (BFA) and saturating concentration of peptide (10 μM). The cells were washed (×4) in X-VIVO 15 plus BFA and then cultured at 37°C. Cells were taken at the indicated times and stained with either anti-D^b^ (KH95) or anti-K^b^ (AF6-88.5) mAbs conjugated with FITC (BD Biosciences). The cells were washed twice and data were collected using a FACSCanto (BD Biosciences) cytometer and analyzed using WinMDI 2.9 software.

### Mice and in vivo experiments

C57BL/6J TRAMP mice were purchased from the Jackson Laboratory (Bar Harbor, ME) and maintained in-house as a hemizygote colony. Genotyping was done by PCR on DNA isolated from ear punches according to the established protocols for the TRAMP transgene 24. Transgenic and non-transgenic littermates were vaccinated at 11–12 weeks of age with a total of 50 μg of plasmid DNA in normal saline injected into two sites in the quadriceps. Evaluation of peptide-specific T-cell responses was performed by ELISPOT analysis as described previously 42. For the GeneSwitch™ experiments, mice were co-injected intramuscularly with 50 μg pCMV-T5/GAL4-T4 and 50 μg pSwitch. At 0 and 6 h after plasmid DNA injection, MFP (Sigma, Poole, UK) was given to the mice intraperitoneally at 5 mg/kg. Animal experiments were conducted according to the UK Home Office license guidelines and approved by the University of Southampton's ethical committee.

### Hybridoma generation

Epitope T4- and T5-specific hybridomas were generated according to Sanderson and Shastri 43. Splenocytes from C57BL/6J mice immunized with pDOM-T4 or pDOM-T5 were restimulated in vitro with the appropriate peptide for 3 days in the presence of 20 U/mL IL-2 before being fused with BWZ.36/CD8α.

### Epitope presentation assay

RMA cells were electroporated with pDOM-T5_A3_T4 MVP constructs using conditions described previously 42. After 20 h, the transfectants were co-cultured with T5- or T4-specific T-cell hybridomas and LacZ activity induced measured at 570 nm using the substrate chlorophenol red-β-d-galactopyranoside (Roche, Mannheim, Germany).

## Abbreviations

IDE: immunodominant epitope
MFP: mifepristone
MVP: MHC variant peptide
DOM: N-terminal domain of Fragment C from tetanus toxin
pMHC: peptide–MHC
SDE: subdominant epitope
Tag: SV40 large T antigen
TRAMP: transgenic adenocarcinoma of the mouse prostate

## Supporting information

Detailed facts of importance to specialist readers are published as ”Supporting Information”. Such documents are peer-reviewed, but not copy-edited or typeset. They are made available as submitted by the authors.
