# Supplementary material for: CD8+ T-cell cross-competition is governed by peptide–MHC class I stability
Source: Eur J Immunol. 2011 Nov 28;42(1):256–63. doi: 10.1002/eji.201142010 (PMC3744744; doi:10.1002/eji.201142010)
Supplement: Supplementary file 1 [file eji0042-0256-sd1.zip › Pages from 201142010_a.pdf]

European Journal of Immunology

Supporting Information

for

DOI 10.1002/eji.201142010

CD8^{+} T-cell cross-competition is governed by peptide–MHC class I stability

Ian Galea, Jana Stasakova, Melanie S. Dunscombe, Christian H. Ottensmeier, Tim Elliott and Stephen M. Thirdborough
